# A semi-quantitative upconversion nanoparticle-based immunochromatographic assay for SARS-CoV-2 antigen detection

**DOI:** 10.3389/fmicb.2023.1289682

**Published:** 2023-12-11

**Authors:** Hai Ding, Wanying Zhang, Shu-an Wang, Chuang Li, Wanting Li, Jing Liu, Fang Yu, Yanru Tao, Siyun Cheng, Hui Xie, Yuxin Chen

**Affiliations:** ^1^Department of Laboratory Medicine, Nanjing Drum Tower Hospital Clinical College of Nanjing Medical University, Nanjing, Jiangsu, China; ^2^Department of Clinic Nutrition, Nanjing Drum Tower Hospital, The Affiliated Hospital of Nanjing University Medical School, Nanjing, Jiangsu, China; ^3^Polariton Life Technologies Ltd., Soochow, Jiangsu, China

**Keywords:** upconversion nanoparticles, SARS-CoV-2, fluorescence immunochromatography assay, antigen detection, semi-quantitative

## Abstract

The unprecedented public health and economic impact of the coronavirus disease 2019 (COVID-19) pandemic caused by severe acute respiratory syndrome coronavirus 2 (SARS-CoV-2) infection has been met with an equally unprecedented scientific response. Sensitive point-of-care methods to detect SARS-CoV-2 antigens in clinical specimens are urgently required for the rapid screening of individuals with viral infection. Here, we developed an upconversion nanoparticle-based lateral flow immunochromatographic assay (UCNP-LFIA) for the high-sensitivity detection of SARS-CoV-2 nucleocapsid (N) protein. A pair of rabbit SARS-CoV-2 N-specific monoclonal antibodies was conjugated to UCNPs, and the prepared UCNPs were then deposited into the LFIA test strips for detecting and capturing the N protein. Under the test conditions, the limit of detection (LOD) of UCNP-LFIA for the N protein was 3.59 pg/mL, with a linear range of 0.01–100 ng/mL. Compared with that of the current colloidal gold-based LFIA strips, the LOD of the UCNP-LFIA-based method was increased by 100-fold. The antigen recovery rate of the developed method in the simulated pharyngeal swab samples ranged from 91.1 to 117.3%. Furthermore, compared with the reverse transcription-polymerase chain reaction, the developed UCNP-LFIA method showed a sensitivity of 94.73% for 19 patients with COVID-19. Thus, the newly established platform could serve as a promising and convenient fluorescent immunological sensing approach for the efficient screening and diagnosis of COVID-19.

## 1 Introduction

Severe acute respiratory syndrome coronavirus 2 (SARS-CoV-2) is the causative agent of coronavirus disease 2019 (COVID-19), which rapidly developed into a global pandemic in 2020 (Jackson et al., 2022). SARS-CoV-2 is an enveloped, positive-sense, single-stranded RNA virus of the genus Betacoronavirus (Lu et al., 2020). Globally, more than 110.7 million cases of SARS-CoV-2 infection and 2.4 million deaths due to COVID-19 were recorded following the outbreak (Rivera et al., 2022). The SARS-CoV-2 infection is characterized by a high transmission rate (transmitted through close person-to-person contact and air by aerosol) and a high mortality rate (Grant et al., 2020; Wang et al., 2021a). The early symptoms (mainly cough and fever) of the SARS-CoV-2 infection are similar to those of infections caused by common respiratory tract viruses, such as influenza A virus (Flu A), influenza B virus (Flu B), and respiratory syncytial virus (RSV) (Cui and Zhou, 2020; Steiner et al., 2020; Wölfel et al., 2020). Therefore, the early diagnosis of SARS-CoV-2 infection facilitates prompt initiation of the specific antiviral treatment.

Presently, nucleic acid-based detection methods, including reverse transcription-polymerase chain reaction (RT-PCR) and metagenomic next-generation sequencing (mNGS), are the gold standard approaches for the clinical diagnosis of SARS-CoV-2 infection (Broughton et al., 2020). Despite their accuracy and sensitivity, the classical nucleic acid-based approaches, including RT-PCR and mNGS, require a long detection period (>2 h), special rooms to avoid contamination, expensive equipment, and trained operators; these limitations hinder their widespread clinical application (Wang et al., 2020). Moreover, although the Ct (cycle threshold) value of the SARS-CoV-2 target gene detected by RT-PCR is a critical indicator to quantify the viral load of clinical samples (Valadan et al., 2022; Ye et al., 2022c; Dip et al., 2023), RT-PCR is a demanding procedure that requires highly skilled clinical laboratory staff and appropriate laboratory sites (Ye et al., 2021, 2022a,b; Zhang et al., 2022).

The serological testing of SARS-CoV-2-specific monoclonal antibodies (mAbs) is also a common detection method; however, this method is limited by false-positive results due to the cross-reactivity of various nucleoproteins from several coronavirus species. Moreover, SARS-CoV-2-specific mAbs are barely detectable at the early stage of infection (Yüce et al., 2021). Therefore, an immunological test for SARS-CoV-2 antigens is a timely and precise approach for COVID-19 diagnosis, as the viral antigens can be detected up to several days before the appearance of clinical symptoms, thereby enabling early detection of the infection (Wang et al., 2021a; Xu et al., 2022). The World Health Organization (WHO) has conditionally approved a batch of SARS-CoV-2 antigen reagents for clinical application (Ye et al., 2022c). However, the currently established SARS-CoV-2 antigen assays, such as lateral flow immunochromatographic assay (LFIA) and enzyme-linked immunosorbent assay (ELISA), have inevitable shortcomings such as inadequate sensitivity (poor for viral concentrations less than 0.1 ng/mL), long detection time, and low repeatability (Li et al., 2019).

To improve the performance of rapid immunoassays, we established an upconversion nanoparticle-based lateral flow immunochromatographic assay (UCNP-LFIA) for the high-sensitivity detection of the SARS-CoV-2 nucleocapsid (N) protein. As an emerging fluorescent nanomaterial for clinical immunological assays (Wang et al., 2004; Chen et al., 2010), UCNPs have remarkable photoluminescent properties, including insensitivity to autofluorescence, with a high potential for application in biosensing, photodynamic therapy, biomedical imaging, and drug delivery, as well as in developing fluorescent probes (Hu et al., 2015; Lan et al., 2016). For example, poly (acrylic acid)-functionalized UCNPs were utilized to form a fluorescent probe, which enabled accurate and sensitive quantification of olaquindox in fish and water samples, with an LOD as low as 1.42 ng/mL (Wen et al., 2023). Additionally, a dual-throughput immunochromatographic test strip based on UCNPs was developed by setting up a bi-directional T-line, realizing simultaneous quantification of ochratoxin A (OTA) and deoxynivalenol (DON) (Chen et al., 2022). In the present study, we first characterized and optimized UCNPs for use in the UCNP-LFIA assay and then determined the analytical characteristics of the assay, such as sensitivity, reproducibility, and recovery rate. Furthermore, pharyngeal swabs collected from SARS-CoV-2-infected individuals were used to validate the developed UCNP-LFIA assay.

## 2 Materials and methods

### 2.1 Materials, reagents, and instruments

The sample pad, conjugate pad, polyvinyl chloride bottom plate, nitrocellulose membrane, and absorption pad were supplied by JiTong Biotechnology Co. (China). HBS-EP + 10X (Catalog #:BR100669, Cytiva, USA), an amino coupling reagent (Catalog #:BR100050, Cytiva), and 10 mM glycine (pH 1.5) (Catalog #:BR100354, Cytiva) were purchased for the antibody affinity test. TEM images were acquired using a Hitachi H-7650 transmission electron microscope at the operating voltage of 120 kV. Nucleic acid test results of COVID-19 patients were analyzed using a Roche 52,410 automated nucleic acid extractor and a Roche 480 fluorescence quantitative PCR instrument.

### 2.2 The preparation of UCNP

UCNPs were supplied by JiTong Biotechnology Co. (China). In brief, a typical procedure is conducted for the synthesis of nanocrystals, such as YCl_3_, Ybl_3_, and ErCl_3_. All chemicals were dissolved in 2 mL of distilled water with vigorous stirring. Subsequently, 6 mL of oleic acid and 15 mL of 1-octadecene were added. The solution was heated to 100°C for 10 min and then to 156°C for 30 min. After cooling to room temperature, 10 mL of methanol solution containing 4 mM of NH_4_F and 2.5 mM of NaOH was added. The mixture was kept at 50°C for 30 min. Once methanol was evaporated, the solution was heated to 300°C under an argon atmosphere for 1.5 h and finally cooled to room temperature. The nanocrystals were precipitated with 10 mL of acetone, collected after centrifugation, and redispersed in 6 mL of cyclohexane.

### 2.3 Determination of binding kinetics for the SARS-CoV-2 N protein and mAbs

SPR analysis was performed to determine the binding affinity of the N-specific mAbs (Catalog #: RM3165-00 and RM3166-00, Vazyme Biotech Co., Ltd., China) and the N protein (Catalog #:CG101-00, Vazyme Biotech Co. Ltd.). The antigen was diluted to 200 nM with 10 mM PBS (pH 7.4, containing 428 mM NaCl +0.005% T-20), and serial three-fold dilutions were performed until a concentration of 2.46 nM was achieved. The flow rate was set to 50 μL/min. The diluted antigen was injected into the flow channel, allowed to bind to N-specific mAbs, coupled to the chip for 180 s, and then subjected to the dissociation process for 600 s. After completion of the dissociation process, the sample was regenerated with 10 mM glycine at pH 1.5. The steps of binding, dissociation, and regeneration were repeated for the different concentrations of the antigen. Finally, the data were analyzed using the 1:1 binding model selected by BIA system software.

### 2.4 Semi-quantitative analysis for SARS-CoV-2 detection

To evaluate the detection limit and sensitivity of UCNP-LFIA, sample solutions with different SARS-CoV-2 N protein concentrations from 100 ng/mL to 0.001 ng/mL were prepared by serial dilution of the SARS-CoV-2 N protein. First, 2 μL of mAb-labeled UCNP was added to the binding pad, and the sample solution of each concentration (20 μL) and 80 μL of buffer were vortexed and transferred to the sample pad. After 15 min of chromatography, the fluorescence intensities on the T and C lines were detected using a fluorescence test strip reader, and the T/C values were calculated using the system software. The UCNP-LFIA method was performed five times for each concentration of the samples. The T/C value of each concentration was used as the vertical coordinate, and the diluted concentration of the SARS-CoV-2 N protein was used as the horizontal coordinate to plot the standard curve. The LOD of the UCNP-LFIA method was determined according to the formula given by the IUPAC: LOD = Y_blank_ + 3SD, where Y_blank_ is the mean T/C values of the blank group, and SD is the standard deviation (Hu et al., 2017).

### 2.5 Determination of specificity and reproducibility of the UCNP-LFIA-based SARS-CoV-2 antigen detection method

To validate the specificity of our UCNP-LFIA-based SARS-CoV-2 antigen detection method, clinical samples of patients infected with SARS-CoV-2, RSV, MP, RhV, Flu A, Flu B, and ADV were collected from the Nanjing Drum Tower Hospital. The collected pharyngeal swabs were subjected to RT-PCR. The T/C values were obtained for each test group under the same experimental conditions, and each test was repeated three times. To determine the reproducibility of the developed UCNP-LFIA method, 15 tubes containing 500 pg/mL and 100 ng/mL SARS-CoV-2 N protein were tested independently, according to previously described standard experimental procedures. The T/C values of this test set were obtained, and the corresponding SD and coefficient of variation (%) were calculated.

### 2.6 Detection of the SARS-CoV-2 N protein antigen in clinical samples

Studies involving human participants were reviewed and approved by the Ethics Committee of the Nanjing Drum Tower Hospital (approval No. 20222–746) and were conducted in accordance with the Declaration of Helsinki. Written informed consent from the participant’s legal guardian/next of kin was not required in accordance with the national legislation and institutional requirements. As clinical samples, pharyngeal swabs were obtained from healthy volunteers and COVID-19 patients admitted to the Nanjing Drum Tower Hospital, Nanjing, China from 1 January 2023 to 30 January 2023. Positive samples were considered to be those obtained from patients with typical symptoms (mainly cough and fever), showing RT-PCR Ct values of ≤40. Negative clinical pharyngeal swabs were collected from healthy volunteers without the symptoms of fever and with RT-PCR Ct values of ≥40 during the same period. Clinical samples were obtained from 48 COVID-19 patients and 20 healthy volunteers without clinical symptoms to evaluate the clinical detection capability of UCNP-LFIA. Next, 80 μL lysate was added to 20 μL of each sample solution to release the N protein. Subsequently, 100 μL of this mixture was dropped onto the sample pad to detect the T/C values. RT-PCR was also performed for each clinical sample to determine the Ct values of the N gene and ORF1a. The correlation between T/C values measured by UCNP-LFIA and Ct values measured by RT-PCR was analyzed using Spearman’s correlation, and *p* < 0.05 was considered statistically significant. Data were analyzed using GraphPad Prism (version 9.0.1, La Jolla, California, USA^1^).

### 2.7 Comparison with the currently used commercial colloidal gold-based LFIA strips

The performance of colloidal gold-based LFIA strips in the semi-quantitative analysis of SARS-CoV-2 was compared with that of UCNP-LFIA. The commercially available SARS-CoV-2 nucleoprotein (Vazyme) was serially diluted in physiological saline to obtain sample solutions in the concentration range of 100–0.01 ng/mL. Next, a mixture of 25 μL of different concentrations of the sample solution and 75 μL of running buffer was prepared and added to the sample pad. After 15 min, the test strips were photographed using a smartphone under the same light conditions.

## 3 Results

### 3.1 Principle of UCNP-LFIA for the detection of SARS-CoV-2 antigens

**Figure 1:**
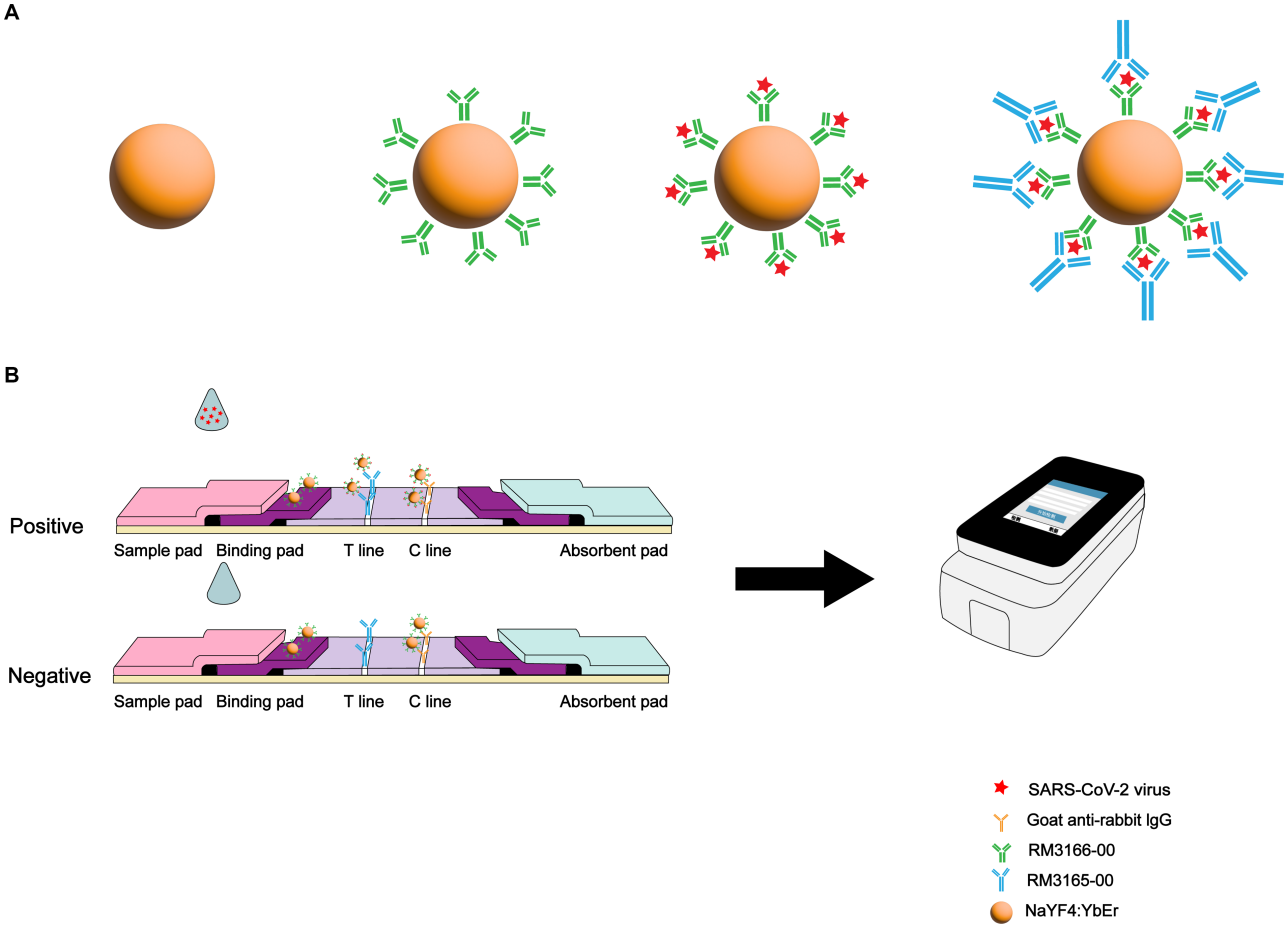
Schematic illustration of SARS-CoV-2 detection using UCNP-LFIA. **(A)** Preparation process for fabricating the mAb-conjugated UCNP. **(B)** Procedure for the detection of SARS-CoV-2 using UCNP-LFIA.

An LFIA biosensor based on SARS-CoV-2 N protein-coupled UCNP was developed for rapid and highly sensitive detection of SARS-CoV-2 infection (Figure 1). First, anti-SARS-CoV-2 N protein mAbs (RM3166)-modified UCNPs were prepared using the ligand exchange method. Next, SARS-CoV-2 N protein detection mAbs (RM3165) and goat anti-mouse mAbs were used for the detection line (T line) and the control line (C line) on the test strip, respectively. A lysate solution was added to the collected specimens in order to release the N protein. The released SARS-CoV-2 N protein was then bound to mAb-modified UCNPs. Following the migration of the solution along the nitrocellulose strip toward the absorbent pad through capillary action, immune complexes were formed on the T and C lines; these complexes produced an intense fluorescence signal that was read by a dedicated upconversion fluorescence reader, and the result was expressed as the ratio of the fluorescence signals on the T and C lines (T/C).

### 3.2 Characterization and optimization of UCNPs

**Figure 2:**
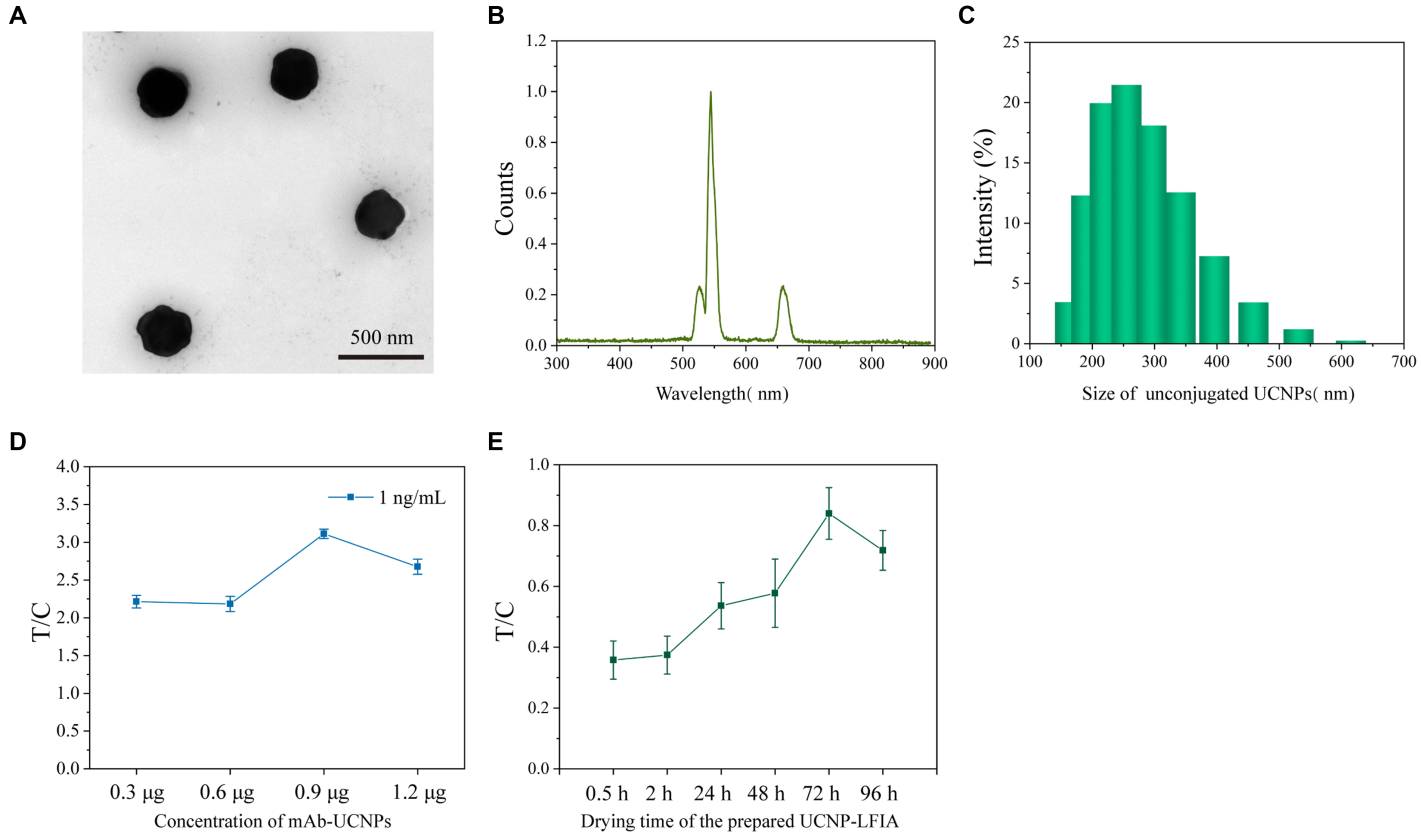
**(A)** TEM images of unconjugated UCNPs. **(B)** The fluorescence spectra of UCNPs. **(C)** DLS distributions of unconjugated UCNPs. **(D)** Optimization of the concentration of mAb-conjugated UCNPs. **(E)** Optimization of the drying time for prepared UCNP-LFIA.

The UCNP used in this study was synthesized by modifying the previously reported method (Wen et al., 2023). The prepared UCNPs were characterized using transmission electron microscopy (TEM). As shown in Figure 2A, the prepared UCNPs were largely spherical with a slight hexagonal tendency. The fluorescence spectrum of UCNPs is shown in Figure 2B, with a strong absorption peak near 540 nm. The results of dynamic light scattering showed that the particle size of UCNPs was relatively homogeneous with an average diameter of 303.3 nm (Figure 2C). The amount of UCNPs deposited on the strip and the drying time of the chromatographic strips were optimized for better assay performance. The conjugate pad was deposited with 0.3, 0.6, 0.9, and 1.2 μg mAb-conjugated UCNPs. The assay was performed with a 1 ng/mL sample. Figure 2D shows the test results. The T/C signal was the highest for 0.9 μg UCNPs. The effect of the drying time of the test strips on the assay was further investigated. The T/C values gradually enhanced with the increase in the drying time, and the best results were achieved for a drying time of 72 h (Figure 2E). The above-mentioned optimized conditions were used for all further experiments.

### 3.3 Binding kinetics of the selected mAb pairs and SARS-CoV-2 N protein

**Figure 3:**
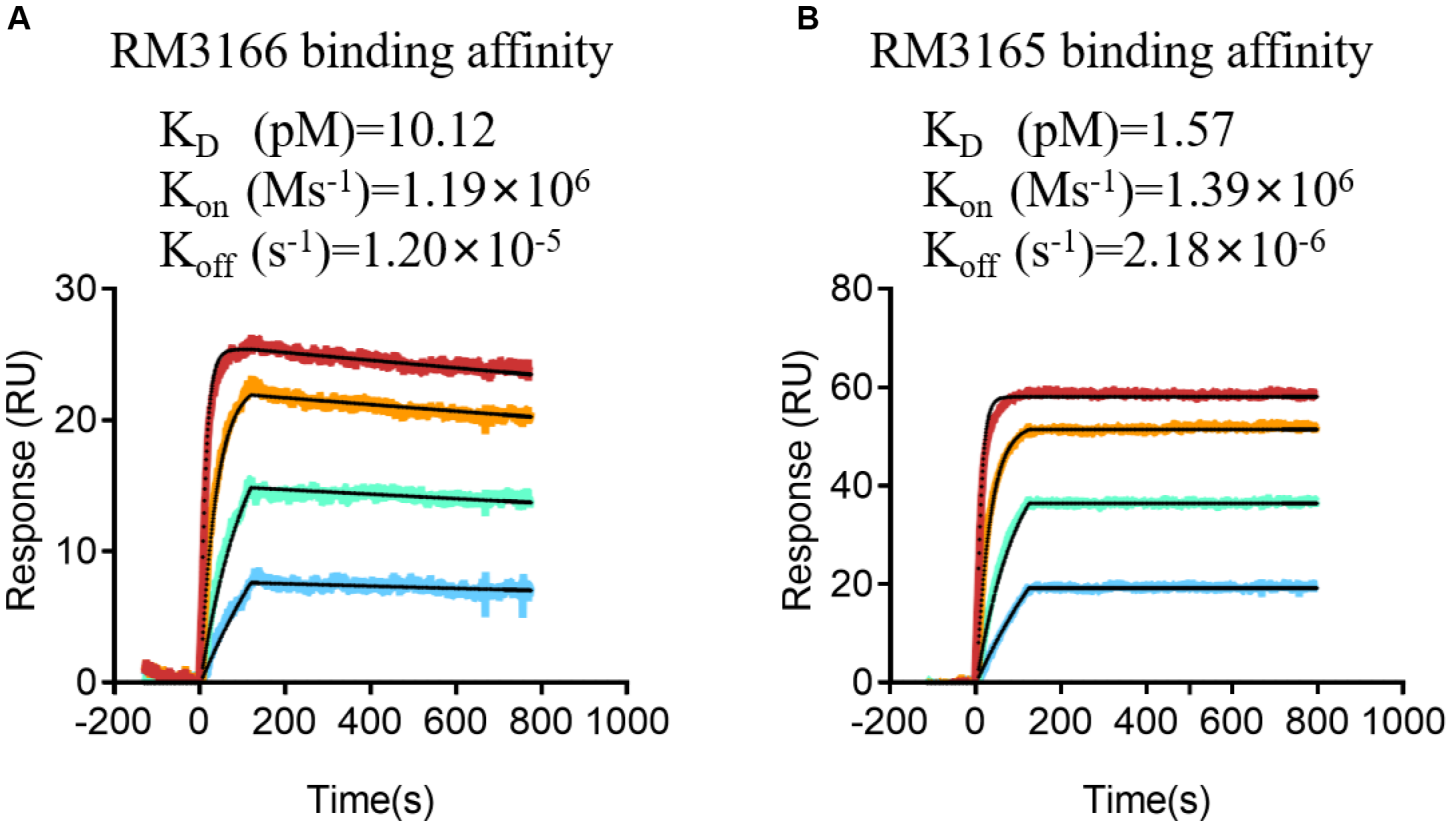
Binding kinetics of capture mAb (RM3166) **(A)** and detection mAb (RM3165) **(B)** with SARS-CoV-2 N protein as measured using Biacore 3,000. Colored lines were original curves, while the black lines were the fitted curves. K_D_ apparent values are shown for the capture or detection of monoclonal mAb binding to the N protein using a 1:1 global fit model.

To determine the binding kinetics of the selected mAb pairs and SARS-CoV-2 N protein, surface plasmon resonance (SPR) analysis was performed using the BIAcore 3,000 instrument. The capture mAb RM3166 showed a binding affinity of 10.12 pM, with a Kon of 1.19 × 10^6^ M^−1^ s^−1^ and Koff of 1.20 × 10^−5^ s^−1^ (Figure 3A), whereas the detection mAb RM3165 showed a binding affinity of 1.57 pM, with a Kon of 1.39 × 10^6^ M^−1^ s^−1^ and Koff of 2.18 × 10^−6^ s^−1^ (Figure 3B).

### 3.4 Sensitivity of UCNP-LFIA for detecting the SARS-CoV-2 N protein

**Figure 4:**
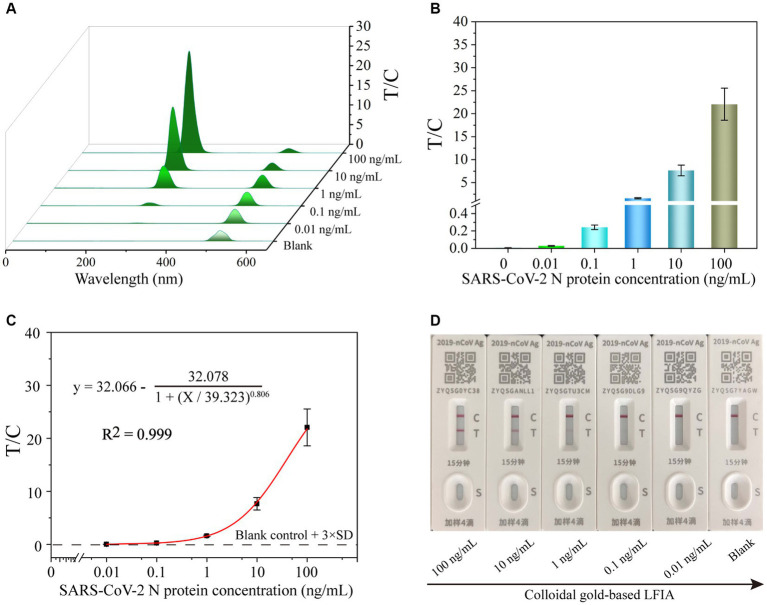
The fluorescence signal **(A)** and corresponding test line intensities **(B)** of UCNP-LFIA between the T/C values and the SARS-CoV-2 N protein antigen detection. **(C)** Corresponding calibration curve for detecting SARS-CoV-2 N protein antigen with the UCNP-LFIA. **(D)** The representative results of colloidal gold-based LFIA detection of SARS-CoV-2 N protein at different concentrations ranging from 0 to 100 ng/mL with 10-fold serial dilution.

To perform the quantitative detection of UCNP-LFIA-based SARS-CoV-2 N protein, the N protein was 10-fold diluted from 100 to 0.001 ng/mL. Subsequently, various concentrations of the N protein were added to UCNP-LFIA, and the fluorescence intensity on the T and C lines was detected using a portable fluorescence detector. The T/C values of the samples decreased with a decrease in the SARS-CoV-2 N antigen concentration, and a fluorescence signal was detected even when the N protein concentration was as low as 0.01 ng/mL (Figures 4A,B). The cutoff of UCNP-LFIA was 0.0058, which was defined as the average signal intensities of negative samples plus three times the standard deviation (SD). Calibration curves were established by plotting the relationship between the T/C values and SARS-CoV-2 N protein concentrations (Figure 4C), and the limit of detection (LOD) was calculated as 3.59 pg/mL using the International Union of Pure and Applied Chemistry (IUPAC) standard method. The commercial colloidal gold LFIA kits currently available in the market showed an LOD of approximately 1 ng/mL (Figure 4D), which is almost 100-fold higher than that of the currently proposed UCNP-LFIA-based N protein detection method.

### 3.5 Specificity and reproducibility of the UCNP-LFIA-based detection method

**Figure 5:**
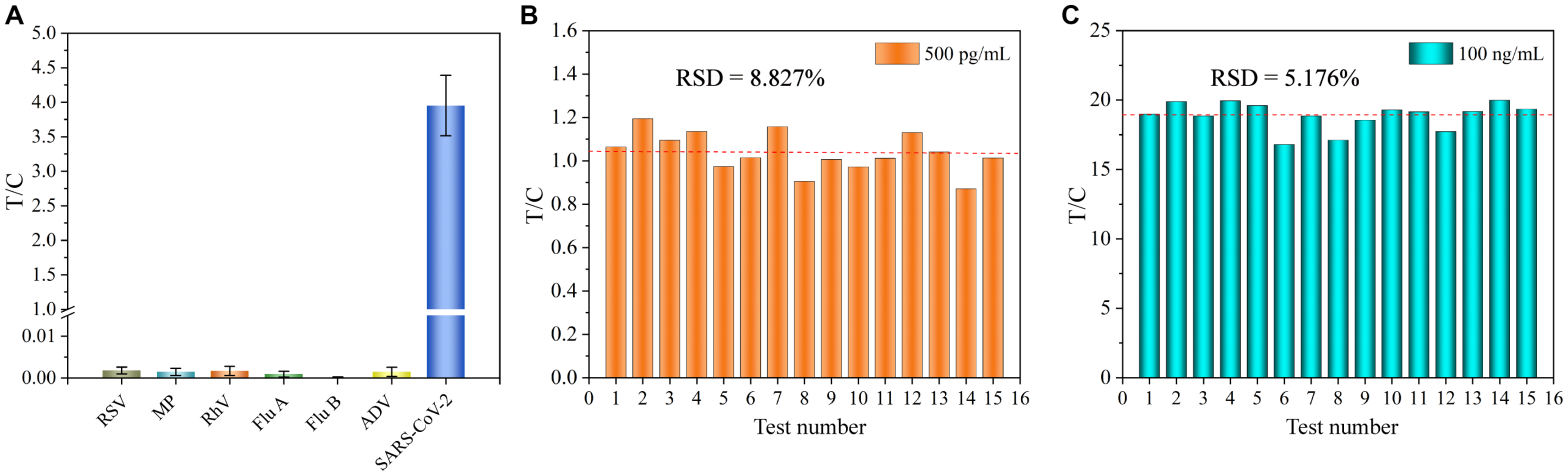
**(A)** Specificity of UCNP-LFIA. Reproducibility of UCNP-LFIA at 500 pg/mL **(B)** and 100 ng/mL **(C)** for 15 random assays.

The specificity of UCNP-LFIA was assessed by testing clinical samples containing other respiratory tract infection-causing microorganisms, including RSV, *Mycoplasma pneumoniae* (MP), rhinovirus (RhV), Flu A, Flu B, and adenovirus (ADV). The T/C values of RSV, MP, RhV, Flu A, Flu B, and ADV were 0.0018, 0.00146, 0.0017, 0.00094, 0.00012, and 0.00146, respectively. In the present study, the T/C value of <0.0058 for the other respiratory tract microorganisms was considered negative. As shown in Figure 5A, no significant T/C values were detected for these interfering strains; this finding was consistent with that for the blank control group. Conversely, significant T/C values were measured in the SARS-CoV-2 group, indicating that the developed UCNP-LFIA method has satisfactory selectivity and specificity for SARS-CoV-2 detection. A series of 15 tests were performed on two sets of samples (500 pg/mL and 100 ng/mL) to further investigate the reproducibility of the method (Figures 5B,C). The relative standard deviation (RSD) for the two sets of samples was estimated as 8.827 and 5.176%, respectively. Thus, our proposed UCNP-LFIA-based SARS-CoV-2 antigen method showed good specificity and reproducibility.

### 3.6 Analytical performance of UCNP-LFIA for clinical samples

**Figure 6:**
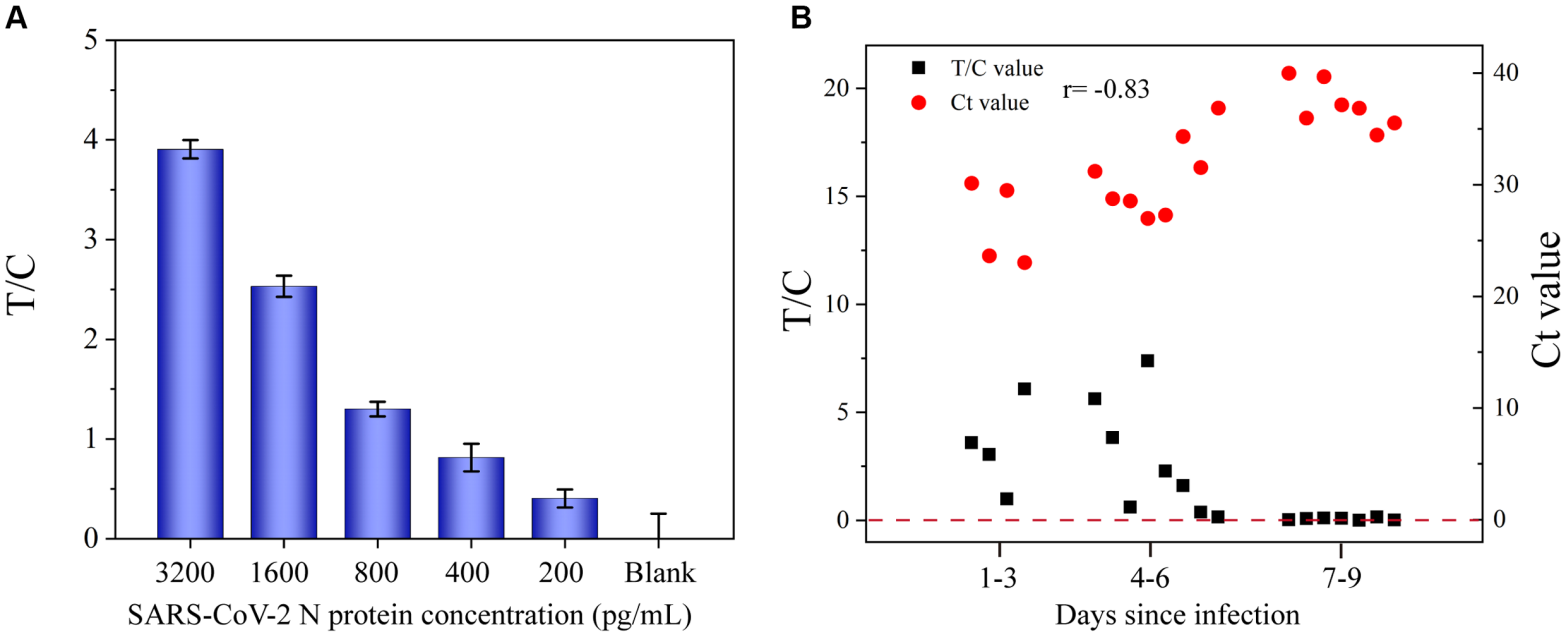
**(A)** Application of UCNP-LFIA to detect the T/C value of SARS-CoV-2 N protein in simulated pharyngeal swab samples. **(B)** Comparison of the T/C values measured by UCNP-LFIA and Ct values measured by RT-PCR in patients with different times of infection.

Different concentrations of the SARS-CoV-2 N protein (200, 400, 800, 1,600, and 3,200 pg/mL) were spiked into the solution of pharyngeal swabs collected from healthy volunteers and tested by the developed UCNP-LFIA method to evaluate its accuracy (Figure 6A). The antigen recoveries corresponding to the five concentrations were 106.3, 117.3, 98.1, 108.3, and 91.1%, respectively (Table 1), with the RSD of <13.9%, indicating that the test strips had acceptable accuracy for the quantification of the SARS-CoV-2 N protein.

**Table 1:** Antigen recovery results of UCNP-LFIA for the SARS-CoV-2 N protein detected in the pharyngeal swab samples.

Samples	Added concentration	Detected concentration	Recovery (%)	RSD (%)
SARS-CoV-2 N protein (pg/mL)	3,200	3402.6	106.3	9.2
	1,600	1876.8	117.3	10.5
	800	784.8	98.1	7.4
	400	433.2	108.3	13.9
	200	182.2	91.1	8.9

To further evaluate the performance of UCNP-LFIA, 68 pharyngeal swab samples collected from patients with typical clinical symptoms and healthy volunteers were subjected to RT-PCR and UCNP-LFIA. Of the 48 specimens collected from COVID-19 patients, 47 specimens tested positive by our proposed UCNP-LFIA method. The sensitivity of UCNP-LFIA detection was 97.73% at the Ct value ≤40 for RT-PCR, and only one specimen with a Ct value of 36.87 for the N gene was diagnosed as negative by UCNP-LFIA. All 20 healthy volunteer samples were tested negative by UCNP-LFIA, which implies a very high negative predictive value (Table 2). As shown in Figure 6B, the T/C values measured by UCNP-LFIA at different time periods of infection were inversely correlated with the Ct values measured by RT-PCR (*r* = −0.83, *p*<0.0001). All samples collected within 6 days of infection showed a positive result for the N protein by our developed UCNP-LFIA method; this finding was consistent with the results of RT-PCR for SARS-CoV-2 nucleic acid. These results validated the remarkable clinical potential of the UCNP-LFIA method to diagnose COVID-19 patients, with high sensitivity (Ct value ≤40) and semi-quantitative properties.

**Table 2:** Sensitivity of the established UCNP-LFIA in the clinical samples with different Ct values of RT-PCR.

			RT-PCR test			
		Ct value ≤32	32 ≤ Ct value ≤35	35 ≤ Ct value ≤40	Ct value ≥40	Total
UCNP-LFIA	Positive	26	8	13	0	47
	Negative	0	0	1	20	21
	Total	26	8	14	20	68
	Sensitivity	100%	100%	92.86%	100%	97.92%

## 4 Discussion and conclusion

The persistent spread of SARS-CoV-2, the specific causative pathogen of COVID-19, has become a severe global public health concern (Coronaviridae Study Group of the International Committee on Taxonomy of Viruses, 2020; Zhou et al., 2020). SARS-CoV-2 infection damages almost all organs of the human body, including the lungs, heart, kidneys, liver, and brain, and can even lead to death in severe cases (Tian and Ye, 2020; Ye et al., 2020a,b,c,d; Chen et al., 2021; Han and Ye, 2021; Zhou and Ye, 2021). Following the development of specific vaccines and implementation of immunization programs globally, COVID-19 is currently no longer a pandemic; however, the mortality rate remains high, particularly in vulnerable groups (Mohseni Afshar et al., 2022). Presently, the mortality and hospitalization rates of COVID-19 can be reduced by taking medicines such as molnupiravir, fluvoxamine, and Paxlovid at the early stage of infection (Singh et al., 2022; Wen et al., 2022; Saravolatz et al., 2023); hence, it is important to perform rapid diagnosis and home monitoring of COVID-19 patients.

In the present study, we developed a UCNP-based immunochromatographic assay (UCNP-LFIA) for the high-sensitivity detection of SARS-CoV-2 antigens, which is beneficial to improve the accuracy and efficiency of detecting SARS-CoV-2 infection in the point-of-care testing (POCT) area. Our developed method showed excellent sensitivity and high specificity because we utilized an upconversion fluorescence strategy. Upconversion fluorescence is a multiphoton process that leads to an anti-Stokes excitation/emission pattern, and this pattern blocks all the naturally occurring fluorescence (e.g., from the lateral flow membrane, supporting adhesives, and biological sample matrix) to yield zero background interference (Baluschev et al., 2006; Zhou et al., 2015; Peltomaa et al., 2021). Thus, it offers high sensitivity and a wide dynamic detection range. In the UCNP-LFIA method, the nasopharyngeal swab solution needs to be dropped on the prepared commercial test strip, and the quantitative results can be read by a portable fluorescence detector after 15 min. A notable finding of the present study is that the results of the UCNP-LFIA method were consistent with those of the “gold standard” RT-PCR, and the antigen fluorescence signal level was equally significant as the Ct value of RT-PCR for guiding clinical treatment and prognosis. Based on the detection of the SARS-CoV-2 N protein in clinical specimens, we found that this method showed significantly higher sensitivity than the commercially available colloidal gold-based antigen detection kit. We also summarized the sensitivity of previously developed LFIA strips based on different signal probe patterns. Compared with earlier methods, the UCNP-LFIA method showed similar or shorter detection time and higher sensitivity because UCNPs can eliminate the optical background interference caused by autofluorescence and light scattering (Table 3) (Liu et al., 2020; Wang et al., 2021b; Han et al., 2022; Oh et al., 2022; Lee et al., 2023; Peng et al., 2023; Wang et al., 2023). Furthermore, in future, this portable fluorescence detector can be developed into a commercial product that can be linked to electronic devices, such as smartphones or smartwatches, to communicate the results to disease control centers or hospitals in time.

**Table 3:** Comparison of UCNP-LFIA with the recently reported LFIA methods for SARS-CoV-2 detection.

Signal mode	Target sample	LODs	Assay time	Ref.
Chemiluminescence-LFIA	S-RBD	100 pg/mL	16 min	Liu et al. (2020)
Fluorescent-LFIA	N protein	5 pg/mL	15 min	Wang et al. (2021b)
Colorimetric/ Fluorescent-LFIA	S1 protein	33 pg/mL	30 min	Han et al. (2022)
Colorimetric-LFIA	N protein	263 pg/mL	15 min	Peng et al. (2023)
Colorimetric-LFIA	N protein	38 pg/mL	10 min	Oh et al. (2022)
Colorimetric-LFIA	S-RBD	630 pg/mL	15 min	Lee et al.(2023)
Fluorescent-LFIA	N protein	8 pg/mL	15 min	Wang et al.(2023)
UCNP-LFIA	N protein	3.59 pg/mL	15 min	This work

S-RBD, receptor-binding domain (RBD) of the SARS-CoV-2 spike S1 protein.

UCNP-LFIA has the advantages of high detection sensitivity, specificity, and reproducibility for SARS-CoV-2 detection within 15 min. Furthermore, the results of our developed UCNP-LFIA method showed good agreement with RT-PCR and a two-fold enhancement in sensitivity as compared with that of the traditional LFIA assay. Considering the significant performance and higher sensitivity of our developed UCNP-LFIA method, we believe that it has a high potential for application in SARS-CoV-2 infection diagnosis in the POCT setting. Despite the many advantages of our proposed method, it still faces some limitations. For example, our proposed algorithm still requires a portable fluorescence detector. Additionally, our current approach could only detect SARS-CoV-2. It would be great if simultaneous detection of common respiratory viruses could be performed.

## Data availability statement

The original contributions presented in the study are included in the article/supplementary material, further inquiries can be directed to the corresponding authors.

## Ethics statement

The studies involving humans were approved by Ethics Committee of Nanjing Drum Tower Hospital (20222–746). The studies were conducted in accordance with the local legislation and institutional requirements. The human samples used in this study were acquired from primarily isolated as part of your previous study for which ethical approval was obtained. Written informed consent for participation was not required from the participants or the participants’ legal guardians/next of kin in accordance with the national legislation and institutional requirements.

## Author contributions

HD: Writing – original draft. WZ: Writing – original draft. S-aW: Writing – original draft. CL: Validation, Writing – review & editing. WL: Formal Analysis, Writing – review & editing. JL: Project administration, Writing – review & editing. FY: Data curation, Writing – review & editing. YT: Data curation, Writing – review & editing. SC: Writing – review & editing. HX: Writing – review & editing. YC: Writing – review & editing.

## Footnotes

^1^ https://www.graphpad-prism.cn/
